# Evaluation of embryo quality after concurrent use of ovarian stimulating hormones and gamma irradiation

**Published:** 2014-08

**Authors:** Tahere Dehghan, Hossein Mozdarani, Arezoo Khoradmehr, Seyed Mehdi Kalantar, Mohsen Bakhshandeh, Fathollah Bouzarjomehri, Seyed Milad Kalantar, Morteza Sepehr Javan

**Affiliations:** 1 *Shahid Beheshti University of Medical Sciences, Tehran, Iran.*; 2 *Department of Medical Genetics, Faculty of Medical Sciences, Tarbiat Modares University, Tehran, Iran.*; 3 *Research and Clinical Center for Infertility, Shahid Sadoughi University of Medical Sciences, Yazd, Iran.*; 4 *Department of Radiology, Shahid Beheshti University of Medical Sciences, Tehran, Iran.*; 5 *Department of Medical Physics, Shahid Sadoughi University of Medical Sciences, Yazd, Iran.*; 6 *Department of Biotechnology, School of Agriculture, Islamic Azad University, Isfahan, Iran.*; 7 *Shahid Sadoughi University of Medical Sciences, Yazd, Iran.*

**Keywords:** *PMSG*-*HCG*, *Gammarays*, *Wholebodyirradiation*.

## Abstract

**Background:** Radiotherapy has many side effects on fertilization in young women. Radiation can lead to ovarian failure in women who underwent abdomen or pelvic radiotherapy.

**Objective:** This study helps us to investigate ovarian response of NMRI female mice to ovarian stimulating hormones (PMSG, HCG) after whole-body gamma irradiation.

**Materials and Methods: **45 pregnant mice were divided into two groups of control and experimental. The experimental group was classified into three sub-groups: Irradiation group (2 or 4Gy),Superovulation group (10 or 15IU),and superovulation and gamma-radiation group (2Gy & 10IU, 2Gy & 15IU, 4Gy & 10IU,4Gy & 15IU). Female mice were killed and embryos were removed from oviduct .The number of embryos cells counted and the quality of them was evaluated in each group. Kruskal-Wallis test and Mann-Whitney test were used to analyze the data.

**Results**: There was a significant difference in the number of 2-4 cells grade D embryos in 2Gy & 15IU group compared with control and 2Gy groups (p=0.01), and the number of embryos in 4Gy group was more than in 10IU and 15IU (p=0.03) and 2Gy & 15IU groups (p=0.01). It was more significantly embryos in 4Gy & 15IU group compared to 2Gy & 15IU group (p=0.01).In addition There were no significant differences in the number of 2-4 cells grades A, B and C embryos and also number of 4-8 cells grades A, B and C, D embryos in groups.

**Conclusion:** The concurrent use of ovulation stimulating hormones and gamma rays ameliorates this problem of drastic decrease in number of living embryos due to whole-body irradiation.

## Introduction

Research advances into cancer treatment improves the quality of life and survival rate in cancer patients. However, side effects of some of these treatment approaches such as radiotherapy on fertility, through affecting the production of sperm cells and gametes and ovary function, should be considered (1-2). In fact, causing trouble in fertility process has been reported as the potential side effect of such a treatment approach. Women who received high doses of pelvic/abdominal radiotherapy or who underwent chemotherapy might have higher risk of infertility (3). The number of ovarian follicles has been drastically reduced by radiation exposure and this process in follicular cells depends on dose and field of radiation. Whole-body irradiation and pelvic radiotherapy can cause permanent ovarian failure (4-7).

Sklar *et al* reported that in patients with cancer for whom both radiotherapy protocols of abdomen and pelvic were used in treating and who underwent chemotherapy, there is a 30% increase in the risk of premature ovarian failure (8). The ovarian granulosa cells are so sensitive to radiation and leading to follicular atrophy is actually an unfavorable side effect of radiation on ovary (9). In a study conducted by Wallace *et al* they showed that older women were at greater risk of damage and their ovaries were more sensitive when radiotherapy was used in the treatment of the cancer. Regardless of the doses and irradiation field, the risk of ovarian failure is higher at abdominal radiotherapy. Wallace *et al* also stated that postnatal oocytes are susceptible to the damage due to radiotherapy. 

They expressed that radiotherapy stops normal cell proliferation and damages cell cycles; radiation can also impairs oocytes because they are extremely vulnerable to radiotherapy and at the risk of undergoing induced apoptosis. In another study, it is showed that in children and young women who underwent radiotherapy with a dose of 20 Gy during 6 sessions, the risk of infertility is about 95% (10). There is a concern that the impact of irradiation on fertility in women of reproductive age is one of the serious side effects caused by radiotherapy. Several options have been suggested to retain fertility in cancer patients. These options are such as ovarian tissue cryopreservation, egg freezing and embryo freezing (11). However, some patients did not use any of these methods before undergoing radiotherapy in order to preserve the chance of their fertility after treatment. Now, after their treatment is done, a question arises: Is there any chance of pregnancy by using ovarian stimulating hormones? 

The aim of this study was to investigate the effect of ovarian stimulating hormones on ovaries of female mice, which got irradiation, and to examine their embryos.

## Materials and methods

This is an experimental study and is conducted in Yazd research and clinical center of infertility in 2013.

**Animals**

For the control group and experimental groups, six to eight-week-old female mice were purchased from animal house of Yazd Research and Clinical Center for Infertility, Iran. Female mice were placed in a separate cage (5 per cage) for at least one week before being used in the experiment. The mice were kept in an environment with 12-hour light and 12-hour dark at the degree of 20-24^o^C and humidity of 60-70%. Mice were fed a standard breeding granulated diet and water ad libitum. After one week, female mice were randomly divided into control group, irradiation group (2 or 4Gy), the group with ovarian stimulating hormones (10 or 15IU), and ovarian stimulating hormones & irradiation group (2Gy and 10IU, 2Gy & 15IU, 4Gy & 10IU, 4Gy, and 15IU).The ethical committee of Yazd Research and Clinical Center for Infertility approved this study (Ref no; 284).

**Irradiation group**

Female mice were exposed to whole body irradiation of a 2 or 4Gy gamma-rays generated from a cobalt-60 source (TheratronII, 780 C, Canada) at a dose of 55c Gy /min, with source sample distance (SSD) =82 cm, field size of 10×10 cm in a room at 23±2^o^C. A 2Gy dose was used because Wallace et al. indicated that the required radiation dose for impairing half of the oocyte cells is less than 4 Gy (LD_50_ <4Gy). And a 4Gy dose was used to examine the effect of higher doses of radiation on ovaries of the mice (12). The female mice were exposed to total body irradiation of 2 or 4Gy gamma ray, in the morning of mating day. In the same evening, each female mouse was made to reproduce with a non-irradiated male mouse and in the following morning, the female mouse was found as a plug-positive mouse, which considered as a sign of pregnancy. On the third day of pregnancy, the ten pregnant mice were examined; five pregnant mice in 2Gy group and five mice in 4Gy group were killed and the embryos were removed from their oviducts by flushing.

**Superovulation group**

Intraperitoneal (IP) injection was applied to female mice in the rate of 10 or 15 international units (IU) of Pregnant Mare’s Serum Gonadotropin Folligon (PMSG; Intervet, Holland) followed by injection of 10 or 15 IU of human chorionic gonadotropin (HCG; Organon, Holland) with 42 to 48- hour intervals. One female mouse was mated with a male mouse for an overnight. The next morning female mice were checked for vaginal plug. On the third day of pregnancy, the ten pregnant mice were examined; five pregnant mice in 10IU group and five mice in 15IU group were killed and the embryos were removed from their oviducts by flushing. 

The act of flushing was done by a syringe (Supa, Tehran, Iran) filled with T6 medium and it was the same and used in all groups. T6 media (ingredients for pH of 7.2-7.4; NaCl (4.73 mg/ml), KCl (110 mg/ml), NaH_2_PO_4_ (50 mg/ml), MgCl_2_.6H_2_O (100 mg/ml), CaCl_2_.2H_2_O (260 mg/ml), NaHCO_3_ (2.10 mg/ml), phenol red (phenolsulfonphthalein or PSP) (10 mg/ml), EDTA (6 mg/ml), glucose (1 mg/ml), and Na-pyruvate (30 mg/ml) were purchased from Sigma; Penicillin G-60 mg/ml and Streptomycin-50 mg/ml (Seromed, Berlin, Germany) and Na-lactate-1.98 mg/ml (Merck). 

The purpose of giving a dose of 15 IU hormone was to study the effect of hormone overdose in mice when the irradiation impaired a great number of ovarian follicles. Therefore, to have identical hormone levels, two different doses of hormone have been applied to both groups of superovulation group and, superovulation and irradiation group.

**Superovulation and irradiation group**

Female mice were exposed to total body irradiation alone at a dose of 2 Gy or 4 Gy approximately 12 to 18 hours prior to HCG injection and 30 to 36 hours after PMSG injection. One irradiated female mouse was kept in a cage with a non-irradiated male mouse for an overnight in order to mate. The next morning female mice were checked for vaginal plug In this group, both doses of 10 IU and 15 IU hormone were used. On the third day of pregnancy, embryos of five pregnant mice, who had received 10 IU hormone and who were exposed to the irradiation of 2 Gy were studied. And another five pregnant mice who had taken 15 IU hormone and the irradiation of 2 Gy, the other five pregnant mice who got 10 IU dose of hormone and the irradiation of 4 Gy and finally the last five pregnant mice who were exposed to 4 Gy irradiation and received 15 IU hormone, were also under investigation.

**Control group**

In this group, there was no treatment. In fact, five female mice were received neither hormone nor irradiation before mating.

**Embryos removal on the third day**

The pregnant female mice were killed by cervical dislocation method, Sixty-four hours after HCG injection. Their oviducts were flushed using a special flushing syringe (Supa, Tehran, Iran) filled with pre-warmed T6 medium under a stereomicroscope (Olympus.SZX16,Japan) to obtain embryos. Embryos of each group were removed. Then, the cells were counted; most of them were 4-8 cell embryos but there were also some embryos with 2-4 cells. The quality of embryo’s cells was also examined. 

**Figure 1:**
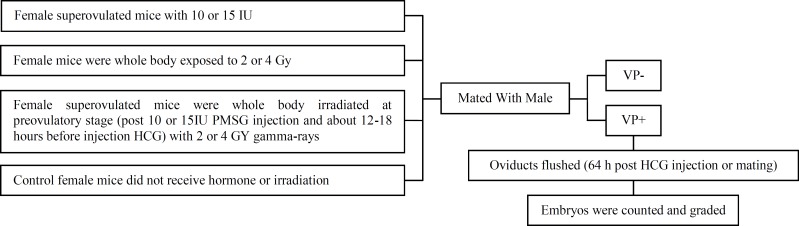
The schematic diagram illustrates the steps performed. Diagram indicates experimental plane used in various stages of the study. VP^+ ^(vaginal plaque positive), VP^¯^ (vaginal plaque negative), PMSG, HCG

The embryos were classified into four grades of A, B, C, and D according to their quality. This kind of embryos’ classification depends on embryos’ appearance which is explained in the following; grade A: cells are of equal size and no fragmentation, grade B: cells are of equal size and minor fragmentation only (10% fragmentation), grade C: cells are of unequal size and no fragmentation to moderate fragmentation (10 to 25% fragmentation), grade D: cells are of equal or unequal size and fragmentation is moderate to heavy (more than 25% fragmentation) (13). All the steps are illustrated in Figure 1.

**Statistical analysis**

Kruskal-Wallis test and Mann-Whitney Test were used for statistical analysis. The whole data were analyzed by SPSS software version 16.0 (Chicago, USA). Statistical significance was set at p<0.05. 

## Results

**Figure 2:**
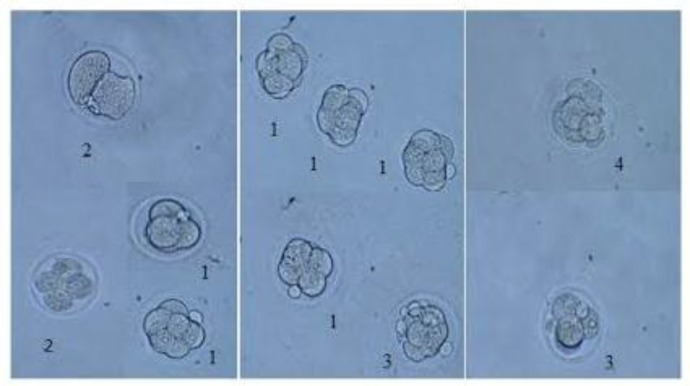
Grading in embryos: 1-grade A 2- grade B 3-grade C A- grade D (x400)

**Figure 3:**
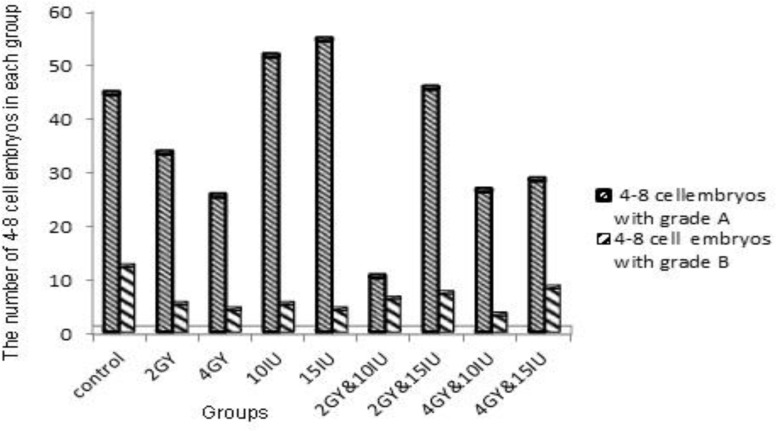
Number of 4-8 cells embryos with grades A, B.

**Figure 4:**
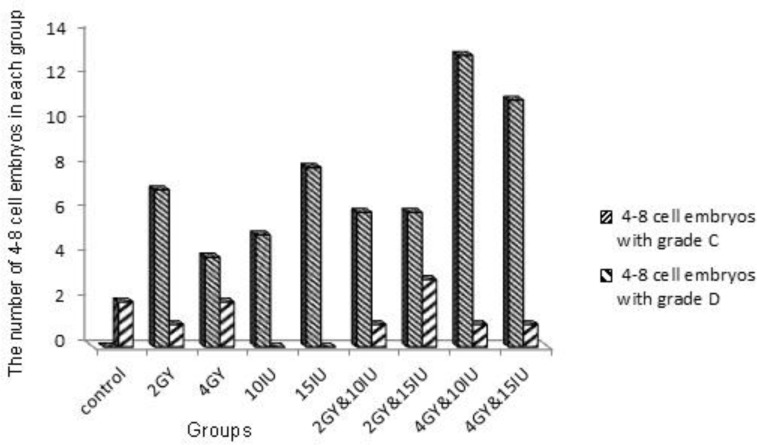
Number of 4-8 cells embryos with grades C, D.

**Figure 5:**
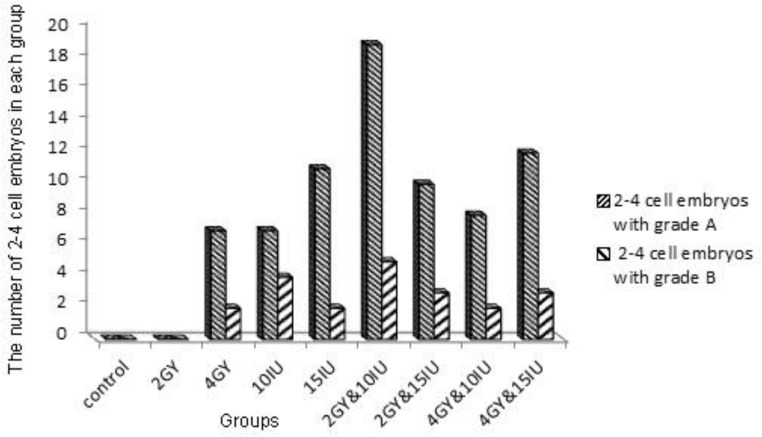
Number of 2-4 cells embryos with grades A, B

**Figure 6:**
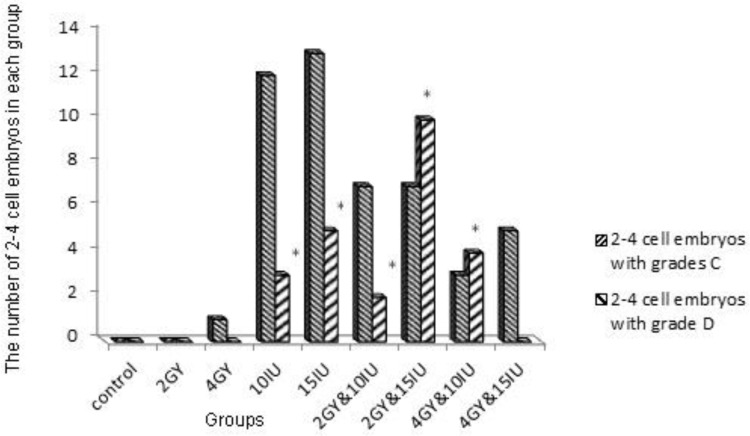
Number of 2-4 cells embryos with grades C, D. (p<0.05*).

The embryos were divided into different quality groups as A, B, C and D higher to lower, respectively (Figure 2). Information about 2-4 and 4-8 cells A, B, C and D embryos has been presented in Figure 3-6. As it is shown in figure 6, the number of 2-4 cells grade D embryos was significantly higher than the number of embryos in other groups (p=0.02). In a comparison between groups, it was indicated that the number of 2-4 cells grade D embryos in 2 Gy &15 group was higher compared with control group and 2Gy group(p=0.01), and it was also significantly higher in 4 Gy group compared with 10 IU and 15 IU group (p=0.03) and 2 Gy &15 group (p=0.01). 

Certainly, it was higher in 4Gy& 15 IU group compared with 2 Gy &15 group (p=0.01, Figure 6). In a comparison between groups, in embryo cell number and embryo quality, there was no significant difference between control group and 15 IU and 2 Gy group. There was also no significant difference in 10 IU group and 15 IU and 2 Gy group. In addition, we got the same result from 15 IU group and 15 IU and 4 Gy group (p=0.05) (Figure 6).

## Discussion

Radiotherapy as a cancer treatment can lead to ovarian failure in patients (14). This side effect depends on many factors such as the patient’s age, pre-treatment patient’s reproductive potential, the total dose and the number of treatments (15). Therefore, when the risk of ovarian failure is being estimated, these factors should be considered. The results of the current study showed that hormone overdose caused decline in the quality of embryos. However, the concurrent use of hormone overdose and irradiation overdose increased the quality of embryos compared with when hormones were used alone. This effect can be caused by the elimination of low-quality ovarian follicles at the high dose of radiation. 

Radiation impairs the primordial and primary follicles and destroys some of the mature follicles (16). Kashchenko *et al* reported that immature follicles are more sensitive to radiation (17). In this study, the results in irradiation group showed that radiation increased the number of 4-8 cells grades C and D embryos (Figure 4). In other studies, it is stated that increase in the dose of radiation in this process, raises the number of poor-quality embryos (18-22).

Ertzeid and Storeng stated that high mortality rate in the embryos of stimulated mice after implantation and low weight in embryos are caused by poor-quality oocytes; and it is caused by the impact of hormones on uterine environment and fetal development (23). According to the other studies, it is concluded that these side effects of ovarian stimulating hormones cause uterine overcrowding, decrease vascular supply for nutrient exchange, and finally decrease the endometrial receptivity. On the other hand, embryos’ assessment before implantation showed that there is a delay in blastocyst formation and fetal growth retardation (24-25). The results in the current study indicated that in superovulation group, hormone can produce more mature and immature follicles (Figure 4). In this study, there was an increase in the number of 4-8 cells grades C and D embryos in 15 IU group compared with the number of 4-8 cells grades C and D embryos in 10 IU group and control group. 

The ovarian stimulating hormone produced more follicles but the follicles had poor quality (Figure 4). The current study tried to explain that the side effect of ovarian stimulating hormone is so related to what was concluded in other studies mentioned before. This study was conducted to prove that among different dose of ovarian stimulating hormones, the high dose did not have a stronger effect but it had negative effects.

In addition to what was concluded from the whole study, various doses of hormone therapy have some negative effects on the quality of embryos and this negative effect could be more in super-high-dose therapy (26-28). In the superovulation and irradiation group, when the mice were exposed to high dose of 4 Gy and received hormone overdose, the number of 4-8 cells grades A and B embryos increased compared with when the mice took a low dose of hormone and were exposed to 2 Gy irradiation. Hormone overdose leads to a great increase in the number of 4-8 cells grade A embryos (Figure 3). 

To conclude, high dose of irradiation has an undesired harmful effect on storage of ovarian follicle, which can cause a decline in the efficiency of ovarian stimulating hormones. But if there was a concurrent use of hormone overdose and high dosage of irradiation, this inefficiency of ovarian stimulating hormones could be improved and the ovary had a higher response and the rate of ovulation could be also higher.

**Suggestions**

The embryos were under investigation for three days, and the focus was on the side effects of radiotherapy and hormone therapy on ovaries whereas both have adverse effects on the uterus. Therefore, there should be a close focus on the following days of pregnancy.This suggestion was extracted from the current research that another study could be conducted in the same condition to investigate the effect of gamma radiation and ovarian stimulation hormones on mice embryos to the end of their pregnancy.

**Limitations**

There were inevitably some limitations in this study. First, because of mice sensitivity to environmental conditions, it was so difficult to keep the condition of animal house constant and to prevent mice mating and pregnancy from being affected by environmental conditions. In addition, according to the effect of mouse age on physiological activity of sex glands (Gonads) in female and male mice and because of age restriction in the current study that we only needed 6 to 8-week-old female mice, observing the age limit was difficult.

## Footnote

***This article extracted from M.Sc. thesis. (Tahereh Dehghan)***
